# SIRT1-modified human umbilical cord mesenchymal stem cells ameliorate experimental peritoneal fibrosis by inhibiting the TGF-β/Smad3 pathway

**DOI:** 10.1186/s13287-020-01878-2

**Published:** 2020-08-18

**Authors:** Yanhong Guo, Liuwei Wang, Rong Gou, Yulin Wang, Xiujie Shi, Xinxin Pang, Lin Tang

**Affiliations:** 1 Department of Nephropathy, The First Affiliated Hospital of Zhengzhou University, No. 1 East Jianshe Road, Zhengzhou, 450052 Henan China; 2 Department of Nephropathy, Henan Provincial Hospital of Traditional Chinese Medicine (The Second Hospital Affiliated to Henan University of Chinese Medicine), No. 6, Dong Feng Road, Jinshui District, Zhengzhou, 450002 Henan China

**Keywords:** Peritoneal fibrosis, SIRT1, hUCMSCs, EMT, TGF-β

## Abstract

**Introduction:**

Peritoneal fibrosis is a serious complication of long-term peritoneal dialysis (PD). Combination therapies are emerging as a promising treatment for tissue damage. Here, we investigated the therapeutic potential of SIRT1-modified human umbilical cord mesenchymal stem cells (hUCMSCs) for peritoneal fibrosis.

**Methods:**

SIRT1 was overexpressed in hUCMSCs to establish SIRT1-modified hUCMSCs. Co-culture and transplantation experiments were performed in TGF-β-stimulated Met-5A cells and peritoneal damage rodent model to assess the therapeutic potential of SIRT1-modified hUCMSCs for peritoneal fibrosis through qPCR, Western blot, and peritoneal function analyses.

**Results:**

SIRT1-modified hUCMSC administration had more potent anti-fibrosis ability than hUCMSCs, which significantly inhibited the expression of fibrotic genes and suppressed EMT process, increased ultrafiltration volume, and restored homeostasis of bioincompatible factors in dialysis solution. Mechanistically, SIRT1-modified hUCMSCs attenuated peritoneal fibrosis through reducing peritoneal inflammation and inhibiting the TGF-β/Smad3 pathway in peritoneal omentum tissues.

**Conclusion:**

SIRT1-modified hUCMSCs might work as a promising therapeutic strategy for the treatment of peritoneal dialysis-induced peritoneal damage and fibrosis.

## Background

Patients with chronic kidney disease gradually lose kidney function; moreover, the incidence and prevalence of this end-stage renal disease (ESRD) rise globally [1]. Peritoneal dialysis (PD) is the common treatment method for patients with ESRD [1, 2]. However, lots of clinical evidence have shown that long-term PD administration often causes the histopathological alteration of the peritoneum and failure of peritoneal function, which is termed as peritoneal damage [2, 3]. The decrease of ultrafiltration capacity, increase of submesothelial fibrosis, and accumulation of extracellular matrix are the common characteristics of peritoneal damage, among them peritoneal fibrosis is the major pathological feature [4, 5]. Various bioincompatible factors in the dialysis solution contribute to the pathogenesis of peritoneal fibrosis, such as high glucose, glycation and osmolality, uremic inflammation, and products of glucose degradation [3, 4, 6, 7]. Hence, exploring the therapeutic approach to alleviate or inhibit peritoneal fibrosis has crucial importance in current research.

Among several therapeutic interventions of peritoneal fibrosis, mesenchymal stem cell (MSC) transplantation has been reported to be promising [8–11]. MSCs are multipotent stromal cells that have regenerative capability and exert paracrine actions on damaged tissues, which can differentiate into a variety of cell types, including osteoblasts, chondrocytes, follicle, myocytes, and adipocytes [12–15]. Recent reports showed that bone marrow-derived MSC transplantation is able to attenuate peritoneal fibrosis and improve peritoneal damage in rat models [16–18]. Wang et al. injected rat bone marrow-derived MSCs into peritoneal scraping induced rat injury model and found MSCs can attenuate peritoneal injury through decreasing inflammation, repairing mesothelial cells, and reducing fibrosis [16]. A group from Japan reported that co-culture rat MSCs and human peritoneal mesothelial cells in the Transwell system can significantly suppress epithelial-to-mesenchymal transition (EMT), downregulate the expression of fibrotic genes in human cells, and finally ameliorate experimental peritoneal fibrosis [17].

EMT plays an important role in many pathological conditions, including tissue fibrosis, pulmonary arterial hypertension, and atherosclerosis [19–21]. The TGF-β signaling pathway is a major cause of abnormal differentiation of EMT-derived fibroblast-like cells, which results in fibrosis in multiple solid organs, such as the liver, lung, heart, and kidney [20, 21]. SIRT1 is a nicotinamide adenosine dinucleotide (NAD)-dependent deacetylase and involved in a broad range of physiological functions, including control of gene expression, cellular reaction to stressors, metabolism, and aging, to exert certain beneficial health effects [22–24]. Huang et al. reported that SIRT1 is an important protective regulator for renal fibrosis in murine mesangial cells and chronic kidney disease rodent model [25]. SIRT1 overexpression reduces TGF-β-induced extracellular matrix expression and production; in contrast, SIRT1 knockdown promotes renal function damage and enhances renal fibrosis [25]. A similar protective effect of SIRT1 was reported by another group, the authors demonstrated that SIRT1 inhibits EMT by deacetylating Smad4 and suppresses the activation of the TGF-β pathway in human endothelial cells [26]. And a combination of stem cell therapies and biotechnology is one of the present promising fields in tissue damage and wound healing. Here, we overexpressed SIRT1 in human umbilical cord mesenchymal stem cells (hUCMSCs) to establish the SIRT1-modified hUCMSC line, investigated the therapeutic effects of SIRT1-modified hUCMSCs on peritoneal fibrosis in cells and rodent model, and clarified the probable underlying mechanism.

## Materials and methods

### Isolation, culture, and identification of hUCMSCs

Fresh human umbilical cords (*n* = 12) were obtained from healthy mothers at Henan Provincial Hospital of Traditional Chinese Medicine hospital following their informed consent. All experimental procedures were approved by the ethics committee of Henan Provincial Hospital of Traditional Chinese Medicine. The hUCMSC isolation was performed by following the MSC isolation protocol created by Cardoso et al. [27]. Isolated cells were cultured in StemXVivo mesenchymal stem cell expansion medium (R&D System, Minneapolis, USA) with l-glutamine (2 mM) and placed in a humidified incubator (37 °C and 8% CO_2_). Cellular morphology was checked every day for 1 week. Cells were expanded when they get 90% confluency, and each passage was harvested and replaced until passage 40. Four cell surface markers (CD34, CD45, CD90, and CD105) were used to determine the hUCMSCs by flow cytometry [28]. Passage 6 hUCMSCs (1 × 10^6^) were stained with anti-CD34, CD45, CD90, and CD105 antibodies (1:500; BD Biosciences, San Diego, USA) for 30 min at room temperature. Flow cytometry (Gilson, Middleton, WI, USA) was used to analyze antibody binding and cell surface markers’ expression as described previously [26].

### Adenoviral plasmids and transduction

The adenoviral constructs of control (pHBAd) and SIRT1 (pHBAd-SIRT1) were kindly provided by Dr. Chen, Shanghai Jiao Tong University School of Medicine, Shanghai. HEK293T cells were used to package and amplify the control and SIRT1 overexpression adenovirus. For transduction, the hUCMSCs (80% confluency) were transfected with control or SIRT1 adenovirus at a multiplicity of infection of 50 and incubated for 4 h. The media were changed and the cells cultured for 2 days, and then the transfected hUCMSCs (SIRT1-modified hUCMSCs) harvested for the subsequent in vitro and in vivo experiments; the control adenovirus-transfected cells (hUCMSCs) were used as control. The effects of SIRT1 adenovirus on the expression of SIRT1 were determined by qRT-PCR and Western blot [24].

### Met-5A culture and treatment

The Met-5A cells (CRL-9444™), an immortalized human pleural mesothelial line, were obtained from the American Type Culture Collection (ATCC, Manassas, USA). Cells were cultured in Gibco Media 199 (Thermo Fisher Scientific, Waltham, USA) with 10% fetal bovine serum (FBS, Gibco, Grand Island, NY) and 2 mM l-glutamine and placed in 37 °C with 8% CO_2_ humidified incubator. The co-culture treatment was conducted in 6-well Transwell (Corning, New York, USA). Met-5A cells were stimulated by TGF-β1 (2.5 ng/mL) for 24 h and then co-cultured with hUCMSCs or SIRT1-modified hUCMSCs (1 × 10^5^ cells) for another 24 h. Met-5A cells were inoculated into the upper compartment, and hUCMSCs or SIRT1-modified hUCMSCs were inoculated into the lower compartment. Whole-cell lysates of Met-5A were collected for the qRT-PCT and Western blot analyses.

### Animals, treatment, and peritoneal equilibration test

Male Wistar rats with 180–220 g body weight were ordered from Shanghai SLAC laboratory Animal Co., Ltd. (Shanghai, China) and used to create the peritoneal fibrosis model by intraperitoneal injection 100 mL/kg of peritoneal dialysate fluid (PDF) with 20 mmol/L MGO, following the protocol established by Li et al. [26]; 8 rats were injected vehicles as the health control. After 2 weeks of PDF daily injection, the rats were divided into three groups (*n* = 8 each group): one group of rats were intravenously injected 1 × 10^7^ hUCMSCs, another group of rats were intravenously injected 1 × 10^7^ SIRT1-modified hUCMSCs, and PBS was injected into the third group as positive control of peritoneal fibrosis. The above intravenous injections were performed one per week for 2 weeks. The peritoneal equilibration test was conducted 1 week after the hUCMSCs/SIRT1-modified hUCMSC treatment. Briefly, anesthetized rat was intraperitoneally injected 18 mL 2.5% PDF in the peritoneal cavity. Two hours after the injection, all the dialysate in the peritoneal cavity was collected by a midline incision. And then the whole blood sample was collected through cardiac puncture. The dialysate creatinine concentration (D), plasma creatinine concentration (P), PDF glucose concentration (D0), and dialysate fluid glucose concentration (D2) were measured by using the fully automatic biochemistry analyzer (Labomed, Inc., Los Angeles, USA). The ultrafiltration volume of each rat was measured and calculated as described previously [26]. The peritoneum of each rat was collected for the subsequent analyses. The study was approved by the ethics committee of Henan Provincial Hospital of Traditional Chinese Medicine.

### Quantitative real-time PCR (qRT-PCR)

The treated Met-5A cells and frozen peritoneum samples were lysed using the TRIzol reagents (Invitrogen, Carlsbad, USA), and total RNA was extracted by using the TRIzol™ plus RNA purification kit (Thermo Fisher Scientific, Waltham, USA). One microgram of total RNA was reversely transcribed into cDNA using the M-MLV reverse transcriptase (New England Biolabs, Ipswich, USA), random primers, and dNTP. The qRT-PCR was conducted by using Bio-Rad CFX96 qPCR system (Bio-Rad, Hercules, USA) with Fast SYBR™ Green Master Mix (Thermo Fisher Scientific, Waltham, USA). The expression levels of tested genes were normalized by human or rat GAPDH and calculated through the 2^-ΔΔCT^ method. Primers used in this study are listed in Table 1.

### Western blotting

Frozen peritoneum samples were lysed by RIPA lysis buffer (150 mM NaCl, 1% NP-40, 50 mM Tris (pH 7.4), 0.1% SDS, 0.5% sodium deoxycholate, and protease inhibitor cocktails). The protein expression levels were detected using Western blotting as previously described [17]. The primary antibody of SIRT1 (ab110304, 1:1000 dilution), TGF-β (ab92486, 1:1000 dilution), α-SMA (ab265588, 1:1500 dilution), Fibronectin (ab268021, 1:2000 dilution), IL-6 (ab233706, 1:1000 dilution), IL-1β (ab234437, 1:1500 dilution), and Smad3 (ab40854, 1:2000 dilution) were purchased from Abcam (Cambridge, UK); pSmad3 (#8769, 1:1500 dilution), TNF-α (#3707, 1:1500 dilution), MCP-1 (#12199, 1:1000 dilution), Calretinin (#17114, 1:2000 dilution), Snail (#3879, 1:1000 dilution), and β-Actin (#4970, 1:3000 dilution) were obtained from Cell Signaling Technology, Inc. (Danvers, USA).

### Histological analysis and immunohistochemistry

The parietal peritoneum of the treated rat was fixed in 4% fresh formaldehyde solution overnight and embedded in paraffin. Masson’s trichrome staining was performed to show the hyperplasia and fibrosis of the parietal peritoneum. Paraffin sections of the parietal peritoneum were used to conduct immunohistochemistry of α-SMA. The primary antibody of α-SMA (1:500, ab5694) was purchased from Abcam (Cambridge, UK). The procedures of immunohistochemistry and intensity of immunostaining (IHC score) calculation were described in the previous study [29].

### Statistical analysis

Statistical analyses were carried out by using the Prism (GraphPad, https://www.graphpad.com/). Student’s *t* test (Tukey’s post hoc test method) was used to analyze the differences between groups. The data were represented as mean ± standard deviation (SD).

## Results

### Characterization of hUCMSCs and SIRT1-modified hUCMSCs

**Fig. 1:**
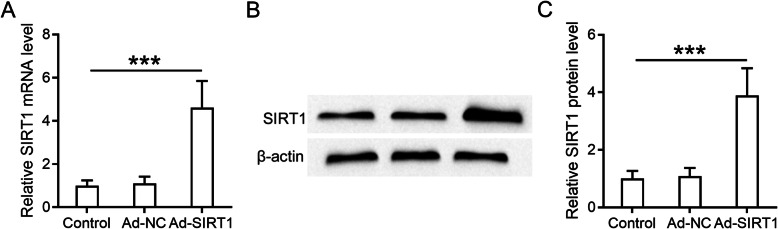
SIRT1 was overexpressed after transfection. **a** hUCMSCs were transfected with Ad-NC or Ad-SIRT1 for 48 h. SIRT1 mRNA expression was detected by qRT-PCR. **b** SIRT1 protein levels were determined by Western blot after transfection, and the relative expressions were normalized to control (**c**). Data are presented as mean ± SD. *n* = 8 for each group. ***p < 0.001 between the indicated groups

After 2-week culture, hUCMSCs reached 90% confluency and formed the swirl colony. Then positive and negative surface markers of MSCs were detected by flow cytometry based on the criteria of MSC identity created by the International Society for Cellular Therapy [26, 28]. The result showed that positive surface markers, CD90 and CD105, are highly expressed on these hUCMSCs, while the negative surface markers, CD34 and CD45, are rarely expressed on the cells (Table 2, control). These results suggested that hUCMSC isolation and culture are successful, and the quality is good for the following experiments. To create the SIRT1-modified hUCMSCs, human SIRT1 was overexpressed in hUCMSCs by adenovirus construct (Ad-SIRT1), and Ad-NC-transfected hUCMSCs were used as the negative control. As shown in Fig. 1a, 48 h after transfection, there was a 5-fold increase of SIRT1 mRNA in hUCMSCs compared to control and Ad-NC groups. Western blot result showed that the protein levels of SIRT1 are significantly elevated (4-fold) after adenovirus-mediated overexpression (Fig. 1b, c). We also performed fluorescence-activated cell sorting of Ad-SIRT1- and Ad-NC-transfected hUCMSCs, in comparison with non-transfected cells; both presented comparable characteristic of MSC surface markers (Table 2).

**Table 1:** Oligonucleotide primer sequences for qRT-PCR

Gene	Primer direction	Sequence (5′–3′)
Fibronectin (human)	Forward	GCTTCCAAGTTGATGCCGTTC
	Reverse	CCGAGCATTGTCATTCAAGGTG
SIRT1 (human)	Forward	AAAGGAATTGGTTCATTTATCAGAG
	Reverse	TTGTGGTTTTTCTTCCACACA
Coll III (human)	Forward	ATGAAGGTGAATTCAAGGCTGAAG
	Reverse	CCACCAATGTCATAGGGTGCAATA
α-SMA (human)	Forward	ATAGAACATGGCATCATCACCAAC
	Reverse	GGGCAACACGAAGCTCATTGTA
Snail (human)	Forward	CCAGACCCACTCAGATGTCAAG
	Reverse	GGGCAGGTATGGAGAGGAAGA
E-cadherin (human)	Forward	CGAGAGCTACACGTTCACGG
	Reverse	GGGTGTCGAGGGAAAAATAGG
GAPDH (human)	Forward	GCACCGTCAAGGCTGAGAAC
	Reverse	TGGTGAAGACGCCAGTGGA
Fibronectin (rat)	Forward	CATCAGCCCGGATGTCAGAA
	Reverse	GGCATTGTCGTTGAGCGTGTA
Coll III (rat)	Forward	TTTGGCACAGCAGTCCAATGTA
	Reverse	GACAGATCCCGAGTCGCAGA
α-SMA (rat)	Forward	AGCCAGTCGCCATCAGGAAC
	Reverse	GGGAGCATCATCACCAGCAA
Snail (rat)	Forward	GCACTGTGATGCCCAGGCTA
	Reverse	CCTTGCCACAGATCTTGCAGAC
Calretinin (rat)	Forward	CACAGACAGAAGTGGCTACATCGAA
	Reverse	TGCCGTCTCCATTTAAGTCAAACA
IL-6 (rat)	Forward	CCCAACTTCCAATG CTCTCCTAATG
	Reverse	GCACACTAGGTTTGCCGAGTAGACC
IL-1β (rat)	Forward	TGTGATGTTCCCATTAGAC
	Reverse	AATACCACTTGTTGGCTTA
TNF-α (rat)	Forward	TCGGTCCCAACAAGGAGGAGAAGT
	Reverse	TGATCTGAGTGTGAGGGTCTGGGC
MCP-1 (rat)	Forward	AGGTCTCTGTCACGCTTCTGGG
	Reverse	TAGCAGCAGGTGAGTGGGGCAT
SIRT1 (rat)	Forward	GAAAATGCTGGCCTAATAGACTTG
	Reverse	TGGTACAAACAAGTATTGATTACCG
TGF-β (rat)	Forward	ATGGTGGACCGCAACAACGCAAT
	Reverse	CAGCTCTGCACGGGACAGCAAT
GAPDH (rat)	Forward	GTATTGGGCGCCTGGTCACC
	Reverse	CGCTCCTGGAAGATGGTGATGG

**Table 2:** Analysis of cell surface marker expressions by flow cytometry after transfection (%, mean ± SD)

Groups (hUCMSCs)	Number	CD105	CD90	CD34	CD45
Control	4	97.86 ± 1.23	98.05 ± 0.89	1.21 ± 0.32	1.98 ± 0.43
Ad-NC	4	96.76 ± 1.62	97.03 ± 2.62	0.96 ± 0.21	1.38 ± 0.39
Ad-SIRT1	4	97.15 ± 0.92	96.61 ± 1.62	1.31 ± 0.62	2.23 ± 0.85
*p* value	–	0.324	0.186	0.294	0.106

hUCMSCs among different groups were analyzed for CD 105, 90, 34, and 45 expressions by using fluorescence-activated cell sorting (FACS) with flow cytometry at passage 6. The negative expressions of CD 34 and 45 and positive expressions of CD 105 and 90 indicated a remaining mesenchymal stem cell lineage after Ad-SIRT1 transfection

### SIRT1-modified hUCMSCs inhibit TGF-β1-induced EMT in vitro

**Fig. 2:**
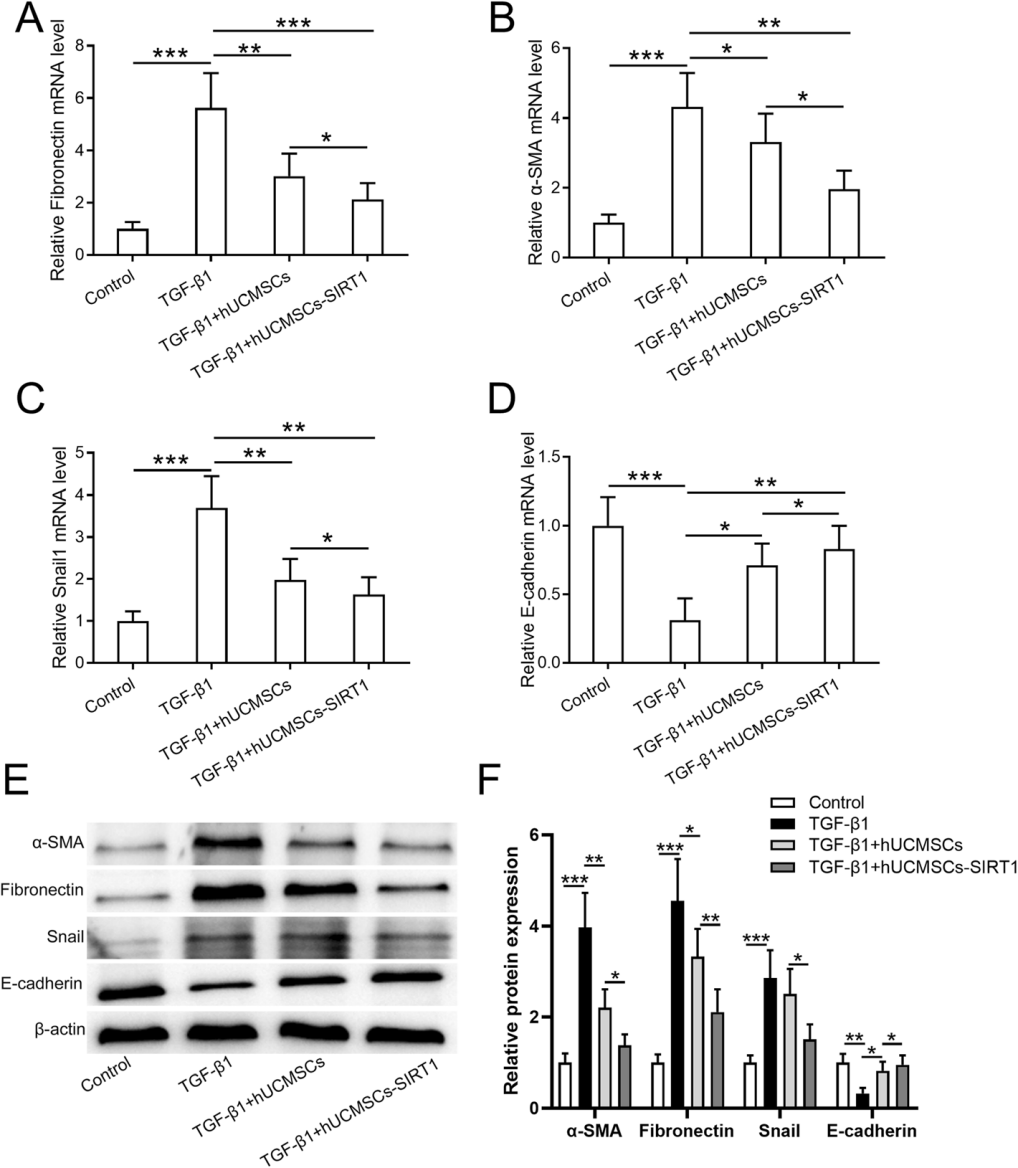
SIRT1-modified hUCMSCs inhibited TGF-β1-induced EMT in Met-5A cells**.** qRT-PCR was used to measure the mRNA levels of Fibronectin (**a**), α-SMA (**b**), Snail (**c**), and E-cadherin (**d**) in the indicated conditions. **e** Western blot was used to measure the protein levels of Fibronectin, α-SMA, Snail, and E-cadherin in the indicated conditions, and the relative expressions were normalized to control (**f**). Data are presented as mean ± SD. *n* = 8 for each group. *p < 0.05, **p < 0.01, and ***p < 0.001 between the indicated groups

Since TGF-β1-induced endothelial-mesenchymal transition is one of the major characteristics of peritoneal fibrosis, we evaluated and compared the protective effects of hUCMSCs and SIRT1-modified hUCMSCs on EMT in the TGF-β1-stimulated Met-5A cells. After co-culture with hUCMSCs and SIRT1-modified hUCMSCs, the EMT status of TGF-β1-stimulated Met-5A cells was determined by the expression of mesenchymal and fibrotic markers, Fibronectin, α-SMA, and Snail. TGF-β1 (2.5 ng/mL) stimulation dramatically increases the expression of Fibronectin, α-SMA, and Snail in Met-5A cells (Fig. 2a–c). Co-culture TGF-β1-stimulated Met-5A cells with hUCMSCs are able to decrease the expression of the above mesenchymal markers in Met-5A cells (Fig. 2a–c). Importantly, co-culture TGF-β1-stimulated Met-5A cells with SIRT1-modified hUCMSCs can further decrease the expression of Fibronectin, α-SMA, and Snail in Met-5A cells relative to the hUCMSC co-culture group (Fig. 2a–c). We also detected the expression of E-cadherin, which is downregulated during EMT [30]. Interestingly, in contrast to mesenchymal markers, the expression of E-cadherin in Met-5A cells is decreased by TGF-β1 stimulation and restored after co-culture with hUCMSCs and SIRT1-modified hUCMSCs; moreover, the strongest induction presents in the SIRT1-modified hUCMSC group (Fig. 2d). Furthermore, the protein level of mesenchymal markers and E-cadherin is consistent with their mRNA changes, and SIRT1-modified hUCMSCs exhibit the strongest inhibitory effects on EMT in the TGF-β1-stimulated Met-5A cells (Fig. 2e, f). These results indicated that SIRT1-modified hUCMSCs significantly inhibit TGF-β1-induced EMT of Met-5A cells.

### SIRT1-modified hUCMSCs attenuate peritoneal fibrosis and peritoneal functional injury

**Fig. 3:**
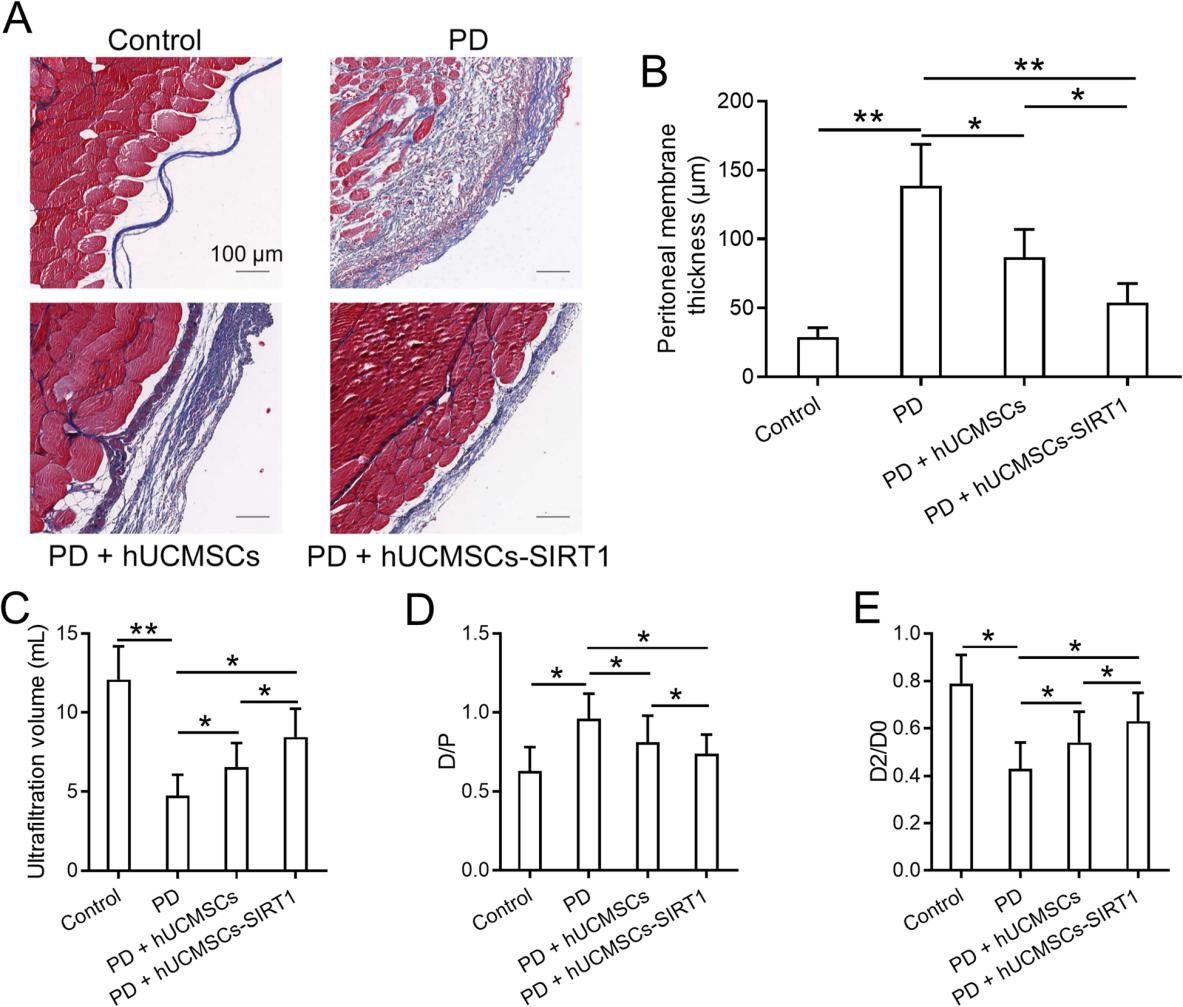
SIRT1-modified hUCMSCs attenuated PD-induced peritoneal fibrosis and peritoneal functional injury. **a** Representative Masson’s trichrome staining of the peritoneum in the indicated groups (bar 200 μm). **b** Mean peritoneal membrane thickness of the submesothelial compact zone in the indicated groups. **c** Ultrafiltration volume in the indicated groups. **d** Dialysate creatinine concentration compared to plasma creatinine concentration from different groups. **e** Dialysate fluid glucose concentration compared to peritoneal dialysate fluid glucose concentration from different groups. Data are presented as mean ± SD. *n* = 8 for each group. *p < 0.05 and **p < 0.01 between the indicated groups

Next, we evaluated the protective effects of SIRT1-modified hUCMSCs on peritoneal damage and fibrosis using the MGO-PD-induced rat model. The parietal peritoneum of the control and treated rats were observed and measured after Masson’s trichrome staining. The staining results showed that PD dramatically increases parietal membrane thickness of the submesothelial compact zone compared to that in the health control rat (Fig. 3a, b). After twice transplantation of hUCMSCs or SIRT1-modified hUCMSCs into the rats, the parietal membrane thickness of the transplanted mice is significantly reduced compared to that in the non-transplanted rat (Fig. 3a, b). Interestingly, transplantation of SIRT1-modified hUCMSCs shows a more potent effect, which is comparable to that in the health control group (Fig. 3b). We further monitored peritoneal function to assess peritoneal injury in the indicated groups through measuring the ultrafiltration volume, ratio of dialysate and plasma creatinine concentration (D/P), and ratio of dialysate and peritoneal dialysate fluid glucose concentration (D2/DO). As shown in Fig. 3c, transplantation of SIRT1-modified hUCMSCs markedly restores the PD-induced reduction of ultrafiltration volume. Moreover, peritoneal equilibration test results showed that transplantation of SIRT1-modified hUCMSCs significantly decreases D/P and increases D2/DO compared to those in the PD-treated control rats (Fig. 3d, e). The above data suggested that SIRT1-modified hUCMSCs attenuate peritoneal fibrosis and restore peritoneal function in PD-induced peritoneal injury.

### SIRT1-modified hUCMSCs alleviate EMT in PD-induced peritoneal damage

**Fig. 4:**
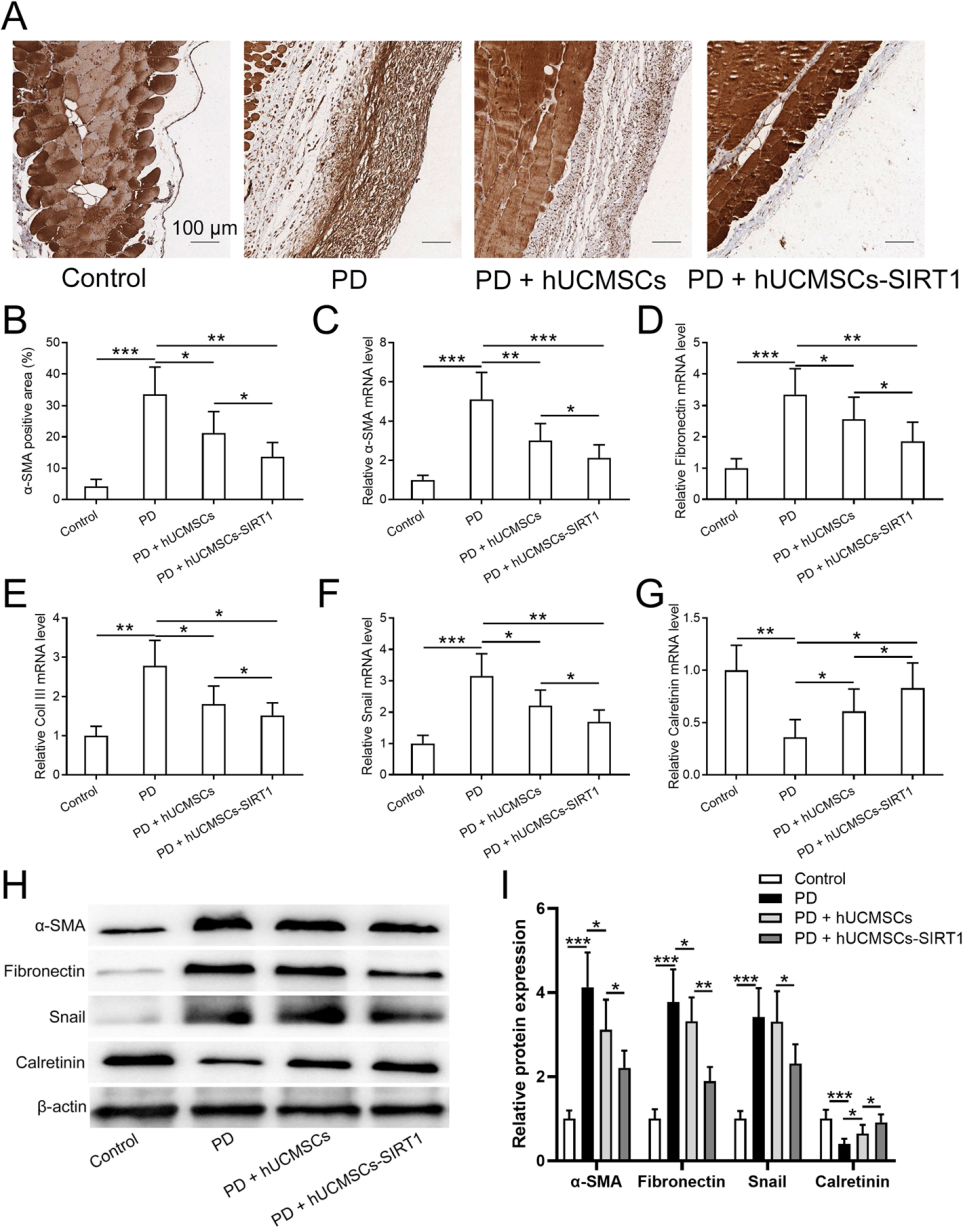
SIRT1-modified hUCMSCs attenuated EMT in PD-induced peritoneal damage. **a** Immunohistochemical analyses of α-SMA expression in peritoneal tissues (bar 200 μm). **b** Accumulation of α-SMA-positive area of **a**. qRT-PCR was used to measure the mRNA levels of α-SMA (**c**), Fibronectin (**d**), Col III (**e**), Snail (**f**), and Calretinin (**g**) in the peritoneal omentum tissues from the indicated groups. **h** Western blot was used to measure the protein levels of Fibronectin, α-SMA, Snail, and Calretinin in the indicated conditions, and the relative expressions were normalized to control (**i**). Data are presented as mean ± SD. *n* = 8 for each group. *p < 0.05, **p < 0.01, and ***p < 0.001 between the indicated groups

We further investigated the protective effects of SIRT1-modified hUCMSCs on EMT in the PD-induced peritoneal damage model. First, we assessed the status of peritoneal fibrosis in the PD-induced injury group and PD plus hUCMSCs or SIRT1-modified hUCMSC transplanted group through detecting the immunohistochemical analysis of α-SMA, a classic fibrotic marker. PD treatment significantly induces the expression and distribution of α-SMA in the peritoneum, while transplantation of hUCMSCs or SIRT1-modified hUCMSCs dramatically inhibits the PD-induced fibrosis; importantly, the SIRT1-modified hUCMSC group exhibits the better inhibitory effect (Fig. 4a–c). Then, we detected the EMT marker genes in the peritoneal omentum tissue of the indicated groups. PD treatment increased the expression of mesenchymal markers, Fibronectin, Col III, and Snail; however, transplantation of SIRT1-modified hUCMSCs significantly suppresses the PD-induced upregulation of these genes (Fig. 4d–f). In contrast, Calretinin, a marker of pleural mesothelial cells, was decreased by PD treatment and restored by SIRT1-modified hUCMSC transplantation (Fig. 4g). In addition, the same trend of mesenchymal markers and Calretinin was observed under the protein level (Fig. 4h, i). All these results suggested that SIRT1-modified hUCMSCs alleviate EMT in the PD-induced peritoneal damage rat model.

### SIRT1-modified hUCMSCs suppress peritoneal inflammation in PD-induced peritoneal damage

**Fig. 5:**
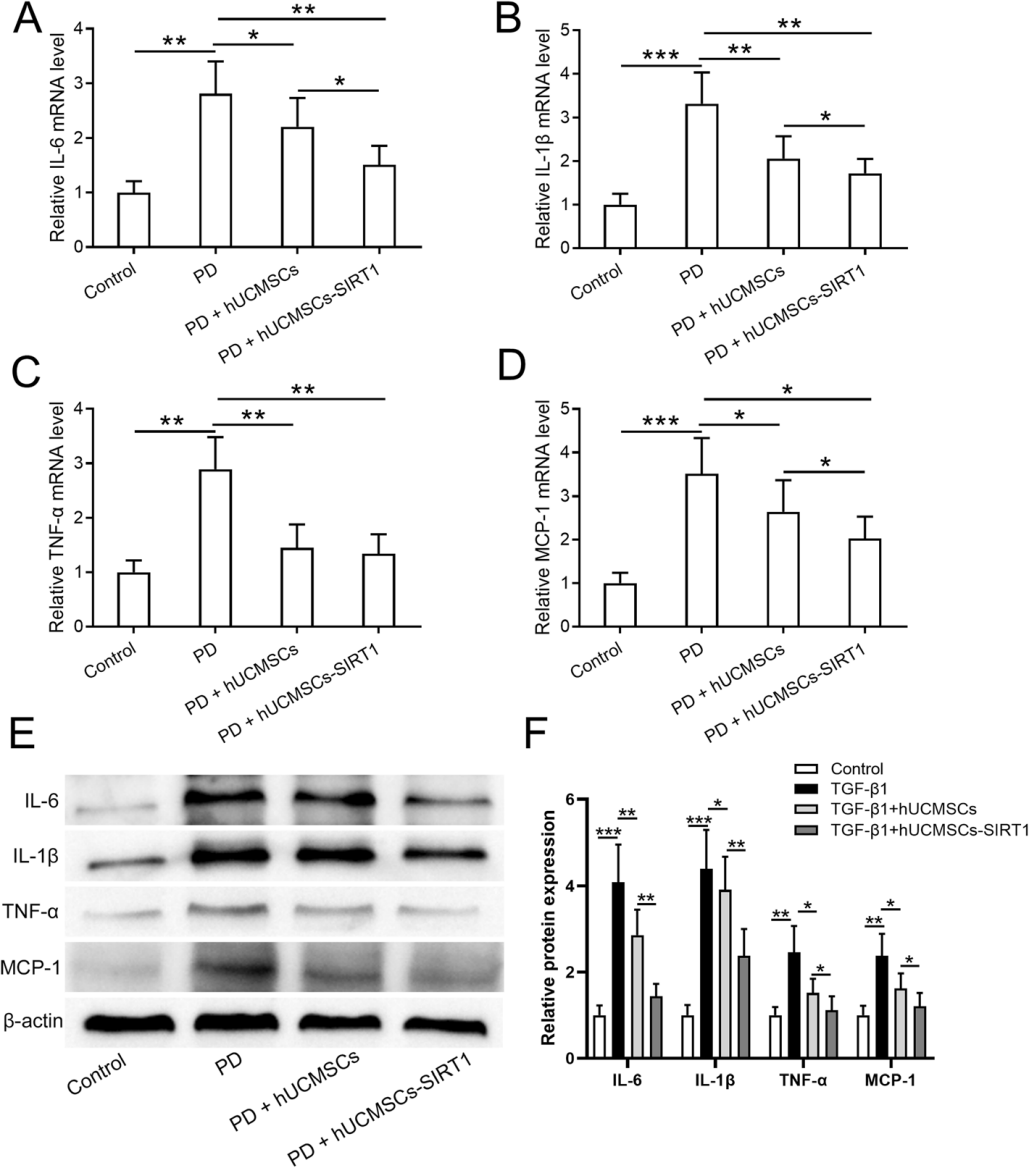
SIRT1-modified hUCMSCs attenuated peritoneal inflammation in PD-induced peritoneal damage. qRT-PCR was used to measure the mRNA levels of IL-6 (**a**), IL-1β (**b**), TNF-α (**c**), and MCP-1 (**d**) in the peritoneal omentum tissues from the indicated groups. **e** Western blot was used to measure the protein levels of IL-6, IL-1β, TNF-α, and MCP-1 in the indicated conditions, and the relative expressions were normalized to control (**f**). Data are presented as mean ± SD. *n* = 8 for each group. *p < 0.05, **p < 0.01, and ***p < 0.001 between the indicated groups

Cytokines and chemokines produced by inflammatory cells and leukocytes play a pivotal role in peritoneal fibrosis and peritoneal functional injury [31]. We examined the peritoneal inflammatory levels in the peritoneal omentum tissues from control, PD-treated, and PD plus hUCMSCs or SIRT1-modified hUCMSC transplantation rat model. As shown in Fig. 5a–d, PD treatment significantly increases the mRNA expression of cytokines, IL-6 (Fig. 5a), IL-1β (Fig. 5b), and TNF-α (Fig. 5c), and chemokine MCP-1 (Fig. 5d) in peritoneal omentum tissues. Transplantation of hUCMSCs or SIRT1-modified hUCMSCs dramatically inhibits the PD-induced elevation of these cytokines and chemokine. Interestingly, in comparison with the hUCMSC group, SIRT1-modified hUCMSC transplantation further lowers the levels of IL-6, IL-1β, and MCP-1 in peritoneal omentum tissues (Fig. 5a, b, d). Moreover, a similar trend of the above cytokines and chemokine was observed by Western blot (Fig. 5e), and the SIRT1-modified hUCMSC transplanted group displays the strongest inhibition of these factors (Fig. 5f). These data suggested that SIRT1-modified hUCMSCs suppress peritoneal inflammation in peritoneal dialysis-induced damage.

### SIRT1-modified hUCMSCs ameliorate peritoneal fibrosis by inhibiting the TGF-β/Smad3 pathway

**Fig. 6:**
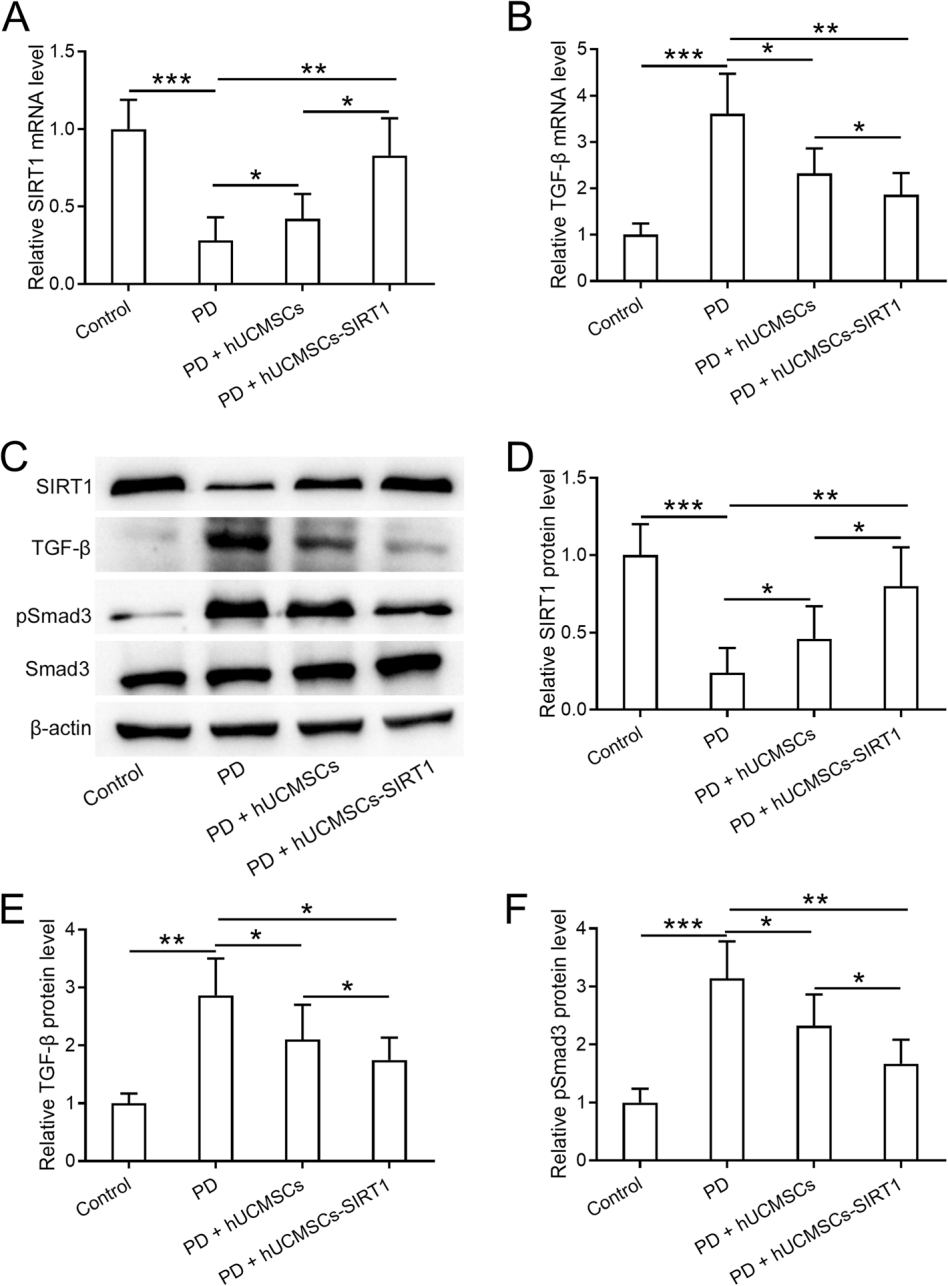
SIRT1-modified hUCMSCs inhibited the TGF-β/Smad3 pathway in PD-induced peritoneal damage. qRT-PCR was used to measure the mRNA levels of SIRT1 (**a**) and TGF-β (**b**) in the peritoneal omentum tissues from the indicated groups. **c** Western blot was used to measure the protein expressions of SIRT1, TGF-β, pSmad3, and Smad3 in the peritoneal omentum tissues from the indicated groups. **d**, **e** The relative expressions were normalized to control. Data are presented as mean ± SD. *n* = 8 for each group. *p < 0.05, **p < 0.01, and ***p < 0.001 between the indicated groups

To explore the underlying molecular mechanism of the protective role of SIRT1-modified hUCMSCs on peritoneal fibrosis, we performed qPCR and Western blot to detect the expression and activity of the TGF-β pathway, which has been reported to induce EMT in human endothelial cells recently [26]. First, we examined the expression levels of SIRT1 and TGF-β in the peritoneal omentum tissues from control, PD-treated, and PD plus hUCMSCs or SIRT1-modified hUCMSC transplantation rats. As shown in Fig. 6a and b, PD treatment decreases the expression of SIRT1 and increases the expression of TGF-β. The SIRT1 expression is restored with the SIRT1-modified hUCMSC transplantation, which significantly inhibits the expression level of TGF-β in the peritoneal omentum tissues (Fig. 6b). We further detected the protein levels of SIRT1 and members of the TGF-β pathway using Western blot and found that PD treatment decreases the expression of SIRT1 protein and increases the protein levels of TGF-β and pSmad3 (Fig. 6c–f). Importantly, both TGF-β and pSmad3 protein levels are significantly reduced with the hUCMSCs or SIRT1-modified hUCMSC transplantation, and the SIRT1-modified hUCMSC group exhibits the stronger inhibitory effect compared to the hUCMSC group (Fig. 6c–f). All these results indicated that SIRT1-modified hUCMSCs ameliorate peritoneal fibrosis which might be through the inhibition of the TGF-β/Smad3 pathway.

## Discussion

Peritoneal fibrosis is a serious complication of long-term peritoneal dialysis, which is considered as an attractive administration for ESRF patients [2, 5, 7]. Therefore, ameliorating or inhibiting peritoneal fibrosis process is an urgent issue that needs to be addressed in basic and clinical fields. Previous studies have shown the evidence that MSCs have the therapeutic effects for the treatment of peritoneal fibrosis through inhibiting EMT in a paracrine manner [26]. SIRT1 is an NAD^+^-dependent deacetylase and plays critical regulatory roles on the self-renewal and multipotency of mesenchymal stem cells [12, 24, 25]. A combination of stem cell therapies and biotechnology is one of the present promising fields in tissue damage and wound healing [32–34]. For example, a combination of adipose-derived MSCs (AD-MSCs) and platelet-rich plasma (PRP) has been used in the treatment of osteoarthritis, progressive joint destruction, and regenerative plastic surgery [35–37]. Human intra- and extra-dermal adipose tissue-derived hair follicle stem cells (HD-AFSCs) have been developed for hair loss treatment through the autologous cellular method [14, 32, 38]. Gentile et al. demonstrated that the combination of adipose-derived stem cells (ASCs) and adipocyte-secreted exosomal microRNA (A-SE-miR) can deliver new anti-cancer molecules in the tumor microenvironment and promote wound repair [14, 15, 39]. In this study, we combined the beneficial effects of MSCs and SIRT1 on the treatment of organ fibrosis to establish the SIRT1-modified hUCMSCs and investigated its therapeutic potential for the treatment of peritoneal fibrosis.

EMT of mesothelial cells plays a key role in the PD-induced organ fibrosis [16, 19, 40]. Patients with long-term PD treatment have increased peritoneal thickness due to the abnormal accumulation and deposition of extracellular matrix, which can be marked by the upregulation of fibrotic and EMT genes [41]. In our research, co-culture SIRT1-modified hUCMSCs with TGF-β-stimulated Met-5A cells significantly inhibit the expression of mesenchymal and fibrotic markers, including Fibronectin, α-SMA, and Snail, and increase the expression of E-cadherin, which has been reported downregulated during EMT [30]. Moreover, transplantation of SIRT1-modified hUCMSCs into peritoneal damage rats dramatically inhibits PD-induced peritoneal membrane thickness and recovers the peritoneal functions. Schilte et al. reported that PD treatment increases glucose absorption, elevates permeability, and reduces ultrafiltration volume; however, heparin administration ameliorates these adverse changes [42]. Another group also demonstrated that hUCMSC treatment increases D2/D0 in MGO-PD-induced peritoneal damage rats [26]. Our results are consistent with the above studies, transplantation of SIRT1-modified hUCMSCs significantly increases the ultrafiltration volume and restores the homeostasis of bioincompatible factors in the dialysis solution. It is worth to point out that SIRT1-modified hUCMSCs have more potent anti-fibrosis ability than the hUCMSCs, indicating SIRT1-overexpressed hUCMSCs possess better therapeutic ability for the treatment of peritoneal fibrosis.

The cumulative evidence has indicated that the TGF-β signaling pathway is an important inducer and regulator in the development and process of peritoneal fibrosis [17, 25, 29, 40]. Adenovirus-mediated TGF-β overexpression in the peritoneum can induce very serious peritoneal fibrosis and neo-angiogenesis, which provided the direct evidence of TGF-β-triggered peritoneal fibrosis [43]. Our findings showed that SIRT1-modified hUCMSC administration attenuates peritoneal inflammation and inhibits the TGF-β/Smad3 pathway in peritoneal omentum tissues. In comparison with hUCMSCs, SIRT1-modified hUCMSCs might improve the paracrine and posttranscriptional modification that have a synergistic effect with hUCMSCs on TGF-β signaling inhibition in peritoneal fibrosis. This is supported by recent findings: SIRT1 physically interacts with SMAD4 and deacetylate SMAD4 in human endothelial cells, which results in the inhibition of TGF-β-induced EMT process and protecting against fibrosis; SIRT1 overexpression attenuate TGF-β-induced extracellular matrix expression in mesangial cells and then ameliorate renal fibrosis [25]. Taken together, this study establishes SIRT1-modified hUCMSC administration as a combination approach with therapeutic potential for the treatment of PD-induced peritoneal damage and peritoneal fibrosis.

Both co-culture and transplantation experiments indicate that the anti-fibrosis effects of SIRT1-modified hUCMSCs act in a paracrine manner, which has been reported in other organ fibrosis studies. Therefore, it is necessary to figure out the SIRT1-modified hUCMSCs secreted effective factors that exert great inhibitory function on EMT and fibrosis in the future study.

## Conclusion

This study investigates the therapeutic potential of SIRT1-modified hUCMSC administration for the treatment of peritoneal fibrosis. The results suggest that SIRT1-modified hUCMSC administration, as a combination approach, has potent therapeutic effects for the treatment of PD-induced peritoneal damage and fibrosis.

## Data Availability

Data could be obtained upon request to the corresponding author.

## Abbreviations

HUCMSCs: Human umbilical cord mesenchymal stem cells
ESRD: End-stage renal disease
PD: Peritoneal dialysis
MSCs: Mesenchymal stem cells
NAD: Nicotinamide adenosine dinucleotide
FBS: Fetal bovine serum
PDF: Peritoneal dialysate fluid
qRT-PCR: Quantitative real-time PCR
SD: Standard deviation
